# Distal humeral plating of an intramedullary nail periprosthetic fracture using a miss-a-nail technique: a case report

**DOI:** 10.1186/1757-1626-2-6704

**Published:** 2009-05-29

**Authors:** Khaled M Sarraf, Ravi Singh, Steven A Corbett

**Affiliations:** 1 Department of Orthopaedic Surgery, Guy's and St Thomas's HospitalLondonUK; 2 Department of Orthopaedic Surgery, Darrent Valley HospitalDarftford, LondonUK

## Abstract

The treatment of distal humeral periprosthetic fractures is not widely described in the literature. We present a difficult clinical scenario of a 72-year-old man who sustained a displaced distal humeral periprosthetic fracture about a Polarus Plus intramedullary nail. In this case, stable fixation was achieved using bicondylar Acumed Mayo congruent Plates using a miss-a-nail technique. Four months following the post operative period, the patient regained satisfactory range of movement with full function and no further complications up to 18 months post fixation. Treatment of such complex periprosthetic fractures is technically achievable and with potentially good results.

## Introduction

Distal humeral fractures associated with previous prosthetic nail present a difficult challenge for both surgeon and patient. The proximity of the fracture to the elbow may further limit the rehabilitation potential as well as the range of movement that has previously been established, thereby compromising the functional outcome [1]. Furthermore, surgical treatment of these fractures may be hindered due to the positioning of the prosthesis and may also be difficult to achieve fracture union [1].

## Case presentation

A 72-year-old right hand dominant retired Caucasian male from Greece presented to the Emergency Department with left upper arm pain. He had sustained a mechanical fall following the consumption of alcohol. His medical history included controlled hypertension, hypercholesterolemia, a smoker of 40 pack years and consumes alcohol in excess of 40 units a week.

**Figure 1.**
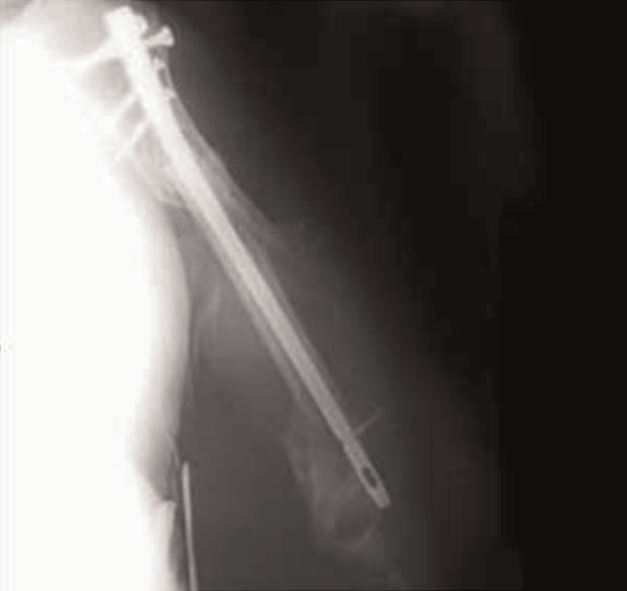
AP radiograph of the periprosthetic fracture of left humerus.

On examination he had a tender, swollen upper arm. The injury was a closed injury with no distal neurovascular deficit. Four years prior to this episode, he had sustained a left segmental humeral fracture requiring stabilization with a Polarus Plus intramedullary humeral nail. Radiographs showed a displaced distal humeral periprosthetic fracture about the Polarus Plus nail with pull out of the distal locking screws (Figure 1).

A posterior approach was undertaken utilising an olecranon osteotomy, with identification and preservation of neurovascular structures. The fractures were identified and circlage wires were used to achieve initial temporary stabilisation prior to definitive fixation. Acumed Mayo medial and lateral plates were used with a combination of cortical and cancellous screws to secure the fractures about the nail. Screws were placed using a miss-a-nail technique, which involves placing screws into the available bone adjacent to an implant to achieve a stable fixation around that implant in order to avoid the original prosthesis. Norian SRS bone graft was used to augment residual bony defect. The Polarus Plus nail was relocked distally using an anterior - posterior locking screw.

**Figure 2.**
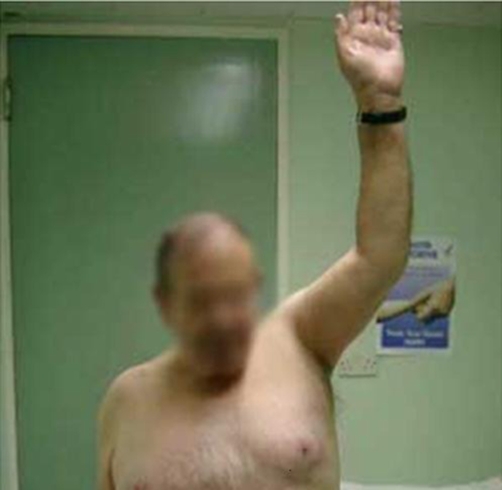
Shoulder flexion (0°-170°).

**Figure 3.**
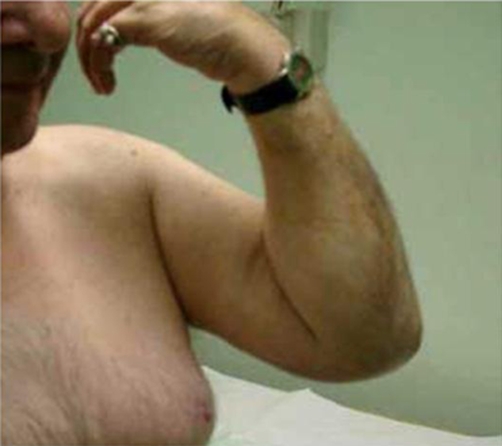
Elbow flexion 150°.

**Figure 4.**
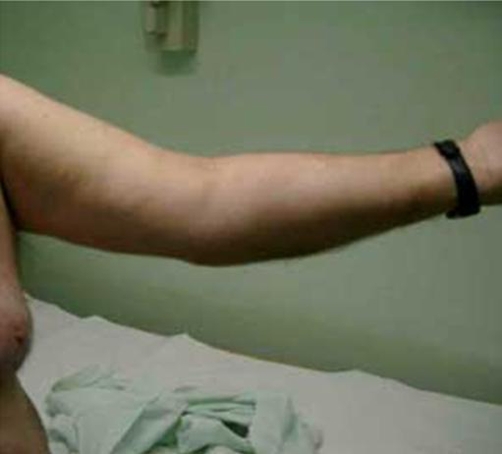
Elbow extension 15°.

At 4 month and up till 18 months post surgery, the patient was pain free. He had active shoulder flexion (0°-170°) (Figure 2), shoulder abduction (0°-150°), external rotation (0°-50°) and elbow flexion (15°-150°) (Figures 3,4).

**Figure 5.**
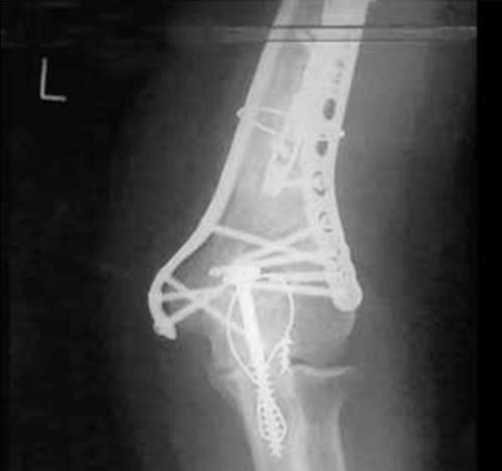
AP radiograph of left elbow post fixation.

**Figure 6.**
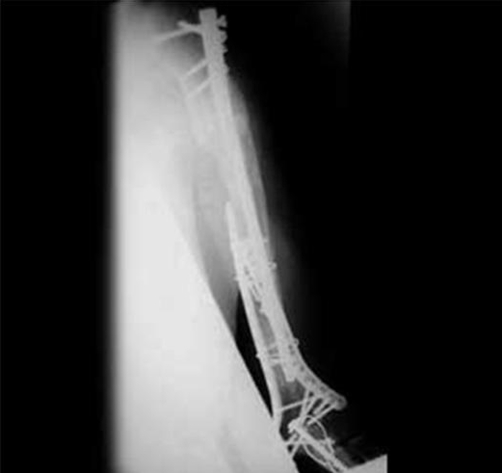
Lateral radiograph of left elbow post fixation with elbow in full extension.

**Figure 7.**
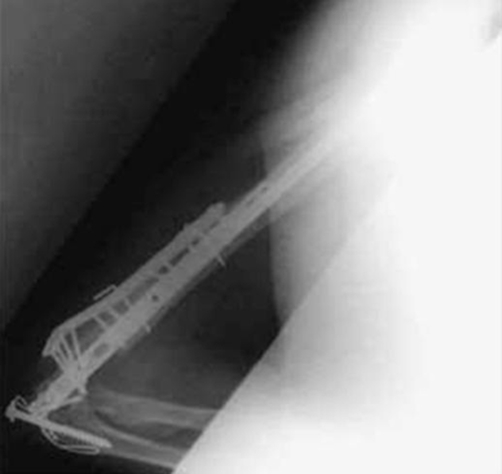
Lateral radiograph of left elbow post fixation with elbow in flexion.

Post operative radiographs showed fractures to have united satisfactorily (Figures 5-7). The patient was very pleased with the outcome, and was able to continue being independent in his activities of daily living.

## Discussion

Fractures about an intramedullary humeral nail have not been frequently described in the literature; most periprosthetic fractures are described in relation to shoulder arthroplasties [1-4]. Postoperative fractures are most commonly caused by minor trauma, such as a fall [5]. Poor bone quality, female sex, advanced age, and history of rheumatoid arthritis are the risk factors most commonly associated with periprosthetic fractures [5]. In this case, the proximity of the fracture to the elbow was concerning and potentially could have limited the rehabilitation potential as well as the range of movement that has previously been established. Thus, using screws secured in a miss-a-nail technique, which has been described in the management of femoral fractures [6,7], and the use of the medial and lateral Mayo plates around the humeral intramedullary nail in situ achieved stability of the periprosthetic fracture. This technique proved to be a successful option with a very good outcome both surgically and functionally for the patient.

## Conclusion

Distal humeral fractures associated with previous prosthetic nail present a challenge to the surgeon to obtain satisfactory results. Leaving the original prosthesis in place and using a miss-a-nail technique with medial and lateral plates around the prosthesis is a good method to secure the fracture with the minimum perioperative trauma to the patient and can achieve a good functional outcome.

## References

[bib-001] Campbell JT, Moore RS, Iannotti JP (1998). Periprosthetic humeral fractures: mechanisms of fracture and treatment options. J Shoulder Elbow Surg.

[bib-002] Worland RL, Kim DY, Arredondo J (1999). Periprosthetic humeral fractures: mechanism of fracture and treatment options. J Shoulder Elbow Surg.

[bib-003] Wright TW, Cofield RH (1995). Humeral fractures after shoulder arthroplasty. J Bone Joint Surg Am.

[bib-004] Boyd AD, Thornhill TS, Barnes CL (1992). Fractures adjacent to humeral prosthesis. J Bone Joint Surg Am.

[bib-005] McDonough EB, Crosby LA (2005). Periprosthetic fractures of the humerus. Am J Othrop.

[bib-006] Jain P, Maini L, Mishra P (2004). Cephalomedullary interlocked nail for ipsilateral hip and femoral shaft fractures. Injury.

[bib-007] Hoffmann R, Sudkamp NP, Muller CA (1994). Osteosynthesis of proximal femoral fractures with the modular interlocking system of unreamed AO femoral intramedullary nail. Initial clinical results. Unfallchirurg.

